# Acids produced by lactobacilli inhibit the growth of commensal *Lachnospiraceae* and S24-7 bacteria

**DOI:** 10.1080/19490976.2022.2046452

**Published:** 2022-03-10

**Authors:** Emma J. E. Brownlie, Danica Chaharlangi, Erin Oi-Yan Wong, Deanna Kim, William Wiley Navarre

**Affiliations:** Department of Molecular Genetics, University of Toronto, Toronto, ON, Canada

**Keywords:** Probiotics, lactobacilli, lactic acid bacteria, *Bacteroidales*, *Clostridiales*, microbiota, gut, acid stress, Lachnospiraceae, Muribaculaceae, S24-7

## Abstract

The *Lactobacillaceae* are an intensively studied family of bacteria widely used in fermented food and probiotics, and many are native to the gut and vaginal microbiota of humans and other animals. Various studies have shown that specific *Lactobacillaceae* species produce metabolites that can inhibit the colonization of fungal and bacterial pathogens, but less is known about how *Lactobacillaceae* affect individual bacterial species in the endogenous animal microbiota. Here, we show that numerous *Lactobacillaceae* species inhibit the growth of the *Lachnospiraceae* family and the S24-7 group, two dominant clades of bacteria within the gut. We demonstrate that inhibitory activity is a property common to homofermentative *Lactobacillaceae* species, but not to species that use heterofermentative metabolism. We observe that homofermentative *Lactobacillaceae* species robustly acidify their environment, and that acidification alone is sufficient to inhibit growth of *Lachnospiraceae* and S24-7 growth, but not related species from the *Clostridiales* or *Bacteroidales* orders. This study represents one of the first in-depth explorations of the dynamic between *Lactobacillaceae* species and commensal intestinal bacteria, and contributes valuable insight toward deconvoluting their interactions within the gut microbial ecosystem.

## Introduction

Lactobacilli are an extensively studied clade of Gram-positive bacteria that are employed in a broad range of applications, including biotechnology, food fermentation, and probiotic formulations.^1–4^ Originally defined as a genus in 1901, this lactic acid-producing group has since expanded to comprise over 260 species based on morphology and fermentation products.^5,6^ However, recent 16S rRNA-based genotyping and genome sequencing efforts have shown that the lactobacilli are far more genetically diverse than most bacterial genera and even many bacterial families.^7,8^ As a result, in 2020 the *Lactobacillus* genus was formally reclassified into 25 genera under the umbrella of the *Lactobacillaceae* family.^6^ This reclassification was carried out using a polyphasic approach predicated on average nucleotide and amino acid identity and core genome phylogeny, while also taking physiology and ecology into consideration.^6^

Members of the newly defined *Lactobacillaceae* family cluster into two distinct clades depending on whether they utilize homofermentative or heterofermentative metabolism.^9,10^ Homofermentative species metabolize hexoses via the Embden-Meyerhof pathway, producing pyruvate as a key metabolic intermediate and lactate as an end product. Heterofermentative species metabolize hexoses via the phosphoketolase pathway, producing pyruvate and acetyl-phosphate as key intermediates with lactate and acetate or ethanol as end products. The split between the two types of metabolism appears to have occurred early in the evolution of the *Lactobacillaceae*, and their fermentation types correlate almost perfectly with phylogeny.^10^

However, beyond this binary metabolic delineation *Lactobacillaceae* species differ with respect to other aspects of their metabolism, physiology, and ecology – occupying niches ranging from free-living to strictly symbiotic.^11^ Importantly, many species from the *Lactobacillaceae* family are resident members of the gut and vaginal microbiota of humans and other animals. This fact, combined with the frequent use of *Lactobacillaceae* species in food and probiotics, has made studying representatives from this family an area of significant focus. Alongside investigating physiological and immunomodulatory effects on the host, over the past few years there has been increased interest in understanding how these species affect other members of the gut microbiota.^12–14^

Considerable research has explored the mechanisms by which specific *Lactobacillaceae* prevent growth of pathogenic bacteria or fungi. From nutrient or niche competition to production of antimicrobial compounds such as bacteriocins, lactic acid, and hydrogen peroxide, a bottom-up approach has been used to characterize the interactions between various *Lactobacillaceae* species and pathogens such as *Listeria monocytogenes, Escherichia coli, Klebsiella pneumoniae, Staphylococcus aureus, and Salmonella enterica*.^14–20^ Fewer studies have examined how *Lactobacillaceae* species interact with commensal gut bacteria, and these have been almost exclusively carried out through a top-down approach by looking at changes in overall bacterial communities. Although this type of work has yielded valuable insights into the dynamics of the gut microbiota, variability in methodology across studies has led to inconsistent findings. For example, some studies have found that probiotic *Lactobacillaceae* species aid in restoring the composition of the gut microbiota after perturbation with antibiotics while others have found that they actively delay reestablishment of the native community.^21,22^ Consequently, elucidating the interactions between specific lactobacilli and the gut microbiota is integral to our understanding of these intricate microbial ecosystems and deciphering their effects on the host.

We recently established the Collection of Inflammation-Associated Mouse Intestinal Bacteria (CIAMIB), which contains isolates from four species of *Lactobacillaceae* (Wong et al., 2022; in press). In exploring the positive and negative interactions between individual isolates within this collection, we found that certain *Lactobacillaceae* species inhibit the growth of bacteria from the *Lachnospiraceae* family (order *Clostridiales*, phylum *Firmicutes*) and the S24-7 group (order *Bacteroidales*, phylum *Bacteroidetes*), while others do not. In the mammalian gut microbiota, the *Firmicutes* and *Bacteroidetes* phyla predominate, and *Clostridiales* and *Bacteroidales* are among the most abundant orders within these phyla. The relative abundance of the *Lachnospiraceae* family in the gut microbiota of mammals is generally above 10%, whereas the abundance of S24-7 bacteria ranges from approximately 2% in the human gut to over 20% in the gut of the laboratory mouse.^23–27^ Despite their prevalence, these taxa remain poorly characterized due to challenges in culturing representative isolates.^25,26,28,29^

Here, we characterized the inhibition of *Lachnospiraceae* and S24-7 isolates by *Lactobacillaceae* utilizing a metabolically diverse set of *Lactobacillaceae* isolates. We discovered that inhibitory activity was common to all homofermentative lactobacilli we tested. We observed that these homofermentative *Lactobacillaceae* species produce higher quantities of acid and allow the pH of the surrounding media to drop, whereas heterofermentative isolates maintain a more neutral environmental pH. Finally, we demonstrated that isolates from the S24-7 group and the *Lachnospiraceae* family are particularly susceptible to acidic conditions compared to related isolates from the *Bacteroidales* and *Clostridiales* orders. This work provides novel insight into the effect of species from the *Lactobacillaceae* family on commensal intestinal taxa and illustrates the importance of using bottom-up approaches to help deconvolute the complex network of interactions within the gut microbiota.

## Results

### *Select species from the* Lactobacillaceae *family inhibit growth of commensal intestinal bacteria in a contact-independent manner*

**Figure 1.**
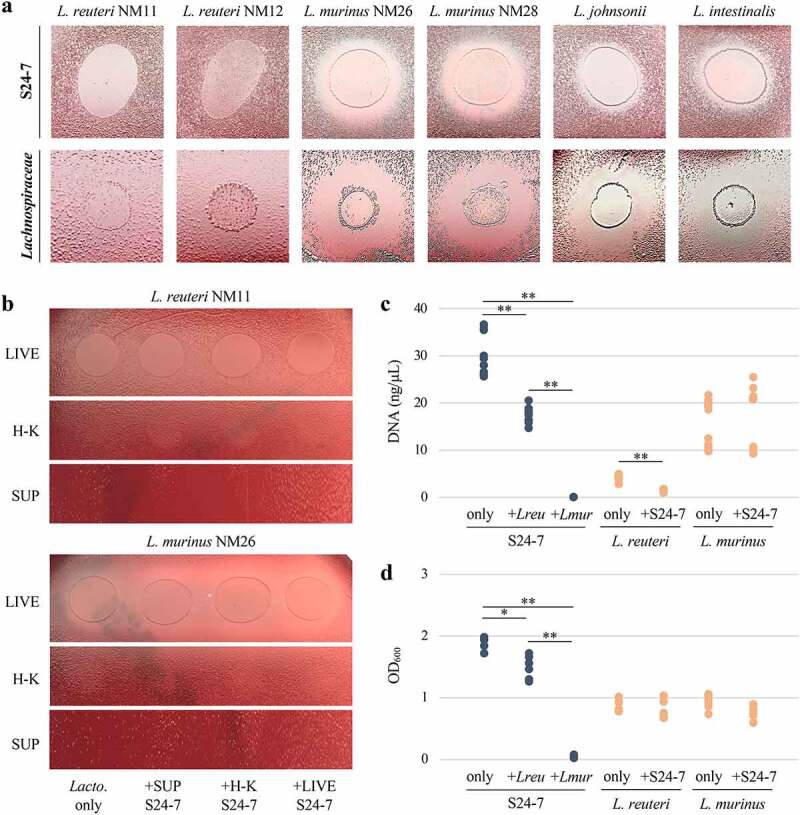
Specific *Lactobacillaceae* species inhibit the growth of representatives from the S24-7 group and *Lachnospiraceae* family in a contact-independent manner. For A) and B), representative images are shown (n = 3). A) Six *Lactobacillaceae* strains spotted onto lawns of NM74_B14 from the S24-7 group and NM01_1-7b from the Lachnospiraceae family. B) Live (LIVE), heat-killed (H-K), and supernatant (SUP) of uninhibitory *L. reuteri* NM11 and inhibitory *L. murinus* NM26 liquid cultures spotted onto NM74_B14. From left to right, spotting is *Lactobacillaceae* grown axenically (*Lacto*. only), with NM74_B14 supernatant (+SUP S24-7), heat-killed (+H-K S24-7), and live (+LIVE S24-7). C) Growth of NM74_B14 (S24-7), *L. reuteri* NM11, and *L. murinus* NM26 measured by qPCR based on DNA concentration. Data points are from four independent experiments. D) Growth measured by OD_600_ of NM74_B14 (S24-7), *L. reuteri* NM11, and *L. murinus* NM26 cultured in transwell plates alone or in the presence of another species. Data points are from three independent experiments. For C) and D), growth of NM74_B14 is shown in blue and growth of *L. reuteri* NM11 and *L. murinus* NM26 is shown in Orange. Welch’s t-test, * p < .05; ** p < .01.

This study began with the exploration of synergistic or antagonistic microbe–microbe interactions using isolates from the CIAMIB. This collection comprises more than 40 strains isolated from the intestines of mice with inflammation and includes four species from the S24-7 group, including isolates from the *Muribaculaceae* family and a newly identified family. The collection also contains isolates of two novel species within the *Lachnospiraceae* family. Initial screening efforts involved spotting assays, where antagonistic interactions between isolates could be observed by the appearance of a zone of growth inhibition in the lawn surrounding the culture drop (Figure 1a). We consistently observed differences between *Lactobacillaceae* isolates in terms of their effect on the growth of isolates belonging to the S24-7 group or the *Lachnospiraceae* family (Table S1). *Ligilactobacillus murinus* (isolates NM26_J9 and NM28_3M-8), *Lactobacillus johnsonii* (isolate NM60_B2-8), and *Lactobacillus intestinalis* (isolate NM61_E11) inhibited the growth of all S24-7 and *Lachnospiraceae* isolates, whereas *Limosilactobacillus reuteri* (isolates NM11_1-41 and NM12_1-47) did not exhibit an inhibitory effect (Figure 1a; Figure S1).

To investigate the mechanism of this inhibition, we performed another set of spotting assays on the S24-7 isolate NM74_B14 and the *Lachnospiraceae* isolate NM01_1-7b, which were selected due to their relatively robust and consistent growth. We sought to determine if growth inhibition required live *Lactobacillaceae* cultures and if the inhibitory activity was stimulated by the presence of S24-7 or *Lachnospiraceae*. The lactobacilli were therefore grown axenically as well as with supernatant, heat-killed bacteria, or live bacteria from the inhibited NM74_B14 or NM01_1-7b isolates. These cultures were then spotted live, heat-killed, or filtered as supernatant onto lawns of NM74_B14 or NM01_1-7b. Only cultures with live *L. murinus, L. johnsonii*, and *L. intestinalis* retained their inhibitory activity. Neither heat-killed bacteria nor filtered *Lactobacillaceae* culture supernatants had an effect on growth (Figure 1b; Figure S2). This suggested that inhibition was either contact-dependent, mediated by a secreted factor that is unstable, or required continual production by living bacteria to reach a concentration necessary to exert its effect.

Using another approach to assess this interaction, we carried out a liquid competition assay where NM74_B14 was co-cultured with uninhibitory *L. reuteri* or inhibitory *L. murinus* and growth of each species was quantified using quantitative PCR (qPCR). Growth of NM74_B14 was completely inhibited in the presence of *L. murinus*, and halved relative to axenic growth when co-cultured with *L. reuteri* (Figure 1c). Notably, growth of *L. reuteri* was also significantly reduced when NM74_B14 was present, while *L. murinus* growth was unaffected by co-culturing. This reciprocal effect on growth of NM74_B14 and *L. reuteri* suggested either nutrient competition or possibly mutualistic behavior between these bacteria. This co-culture assay was not performed with *Lachnospiraceae* as both isolates from the CIAMIB grow poorly in liquid media.

To address whether inhibition was dependent on cell–cell contact, we conducted growth assays using transwell plates containing an insert with a membrane that is impermeable to bacterial cells but permeable to smaller molecules. In these plates NM74_B14 was cultured either alone, across the membrane from uninhibitory *L. reuteri*, or across the membrane from inhibitory *L. murinus*. Given that each species was grown in a separate compartment, turbidity was used to quantify growth. Similar to the qPCR assays, growth of NM74_B14 was decreased by *L. reuteri* and fully inhibited by *L. murinus* (Figure 1d). These results demonstrated that inhibition of S24-7 growth by *L. murinus* does not require cell-to-cell contact and is due to a secreted factor or metabolite.

### *Inhibitory and uninhibitory* Lactobacillaceae *species cluster according to phylogeny and metabolic properties*

The considerable variation in gene content and low protein sequence homology in the genomes of *L. murinus, L. johnsonii, L. intestinalis*, and *L. reuteri* species (Figure S3) made it difficult to infer the cause of the observed growth inhibition from this small set of isolates. To test a larger set of species and catalog the distribution of inhibitory activity within the *Lactobacillaceae* family, we obtained a larger collection of *Lactobacillaceae* isolates selected to encompass a broad range of the genera from this family, amounting to 21 strains from 18 species, in addition to the 6 strains and 4 species from the CIAMIB (Table S2).

Spotting assays were repeated with the expanded panel of *Lactobacillaceae* isolates against NM74_B14 or NM01_1-7b. These assays showed that *Limosilactobacillus vaginalis, Limosilactobacillus oris, Limosilactobacillus coleohominis, Levilactobacillus brevis*, and *Lentilactobacillus parafarraginis* were uninhibitory (Table 1; Figure S4a). Conversely *Ligilactobacillus ruminis, Lactobacillus gasseri, Lactobacillus crispatus, Lactobacillus jensenii, Lactobacillus iners, Lactobacillus psittaci, Lactobacillus delbrueckii, Lacticaseibacillus rhamnosus, Lactiplantibacillus plantarum, Loigolactobacillus coryniformis, Companilactobacillus farciminis, Companilactobacillus alimentarius*, and *Schleiferilactobacillus shenzhenensis* all showed signs of inhibition (Table 1; Figure S4b). For the S24-7 isolate NM74_B14 the zones of clearance were smaller and difficult to detect for *L. jensenii* 269–3, *L. iners, L. psittaci*, and *C. farciminis*. This suggested that S24-7 species are slightly less susceptible to inhibition than *Lachnospiraceae* species. Notably, all isolates of the *Ligilactobacillus, Lactobacillus, Lacticaseibacillus, Lactiplantibacillus, Loigolactobacillus, Companilactobacillus*, and *Schleiferilactobacillus* genera were inhibitory to some extent. No isolates from the *Limosilactobacillus, Levilactobacillus*, and *Lentilactobacillus* genera showed an inhibitory effect on either S24-7 or *Lachnospiraceae*.

**Table 1.** Inhibitory activity of a range of *Lactobacillaceae* species on growth of S24-7 and *Lachnospiraceae* representatives. Plus sign (+) indicates inhibition of NM74_B14 (S24-7) and NM01_1-7b (*Lachnospiraceae*). Minus sign (-) indicates no effect on growth of either isolate

Genus	Species	Strain	Inhibitory activity
***Limosilactobacillus***	*L. reuteri*	NM11	-
		NM12	-
	*L. vaginalis*	EX336960VC11	-
	*L. oris*	F0423	-
	*L. coleohominis*	DSM14060	-
***Levilactobacillus***	*L. brevis*	DSM20054	-
***Lentilactobacillus***	*L. parafarraginis*	DSM18390	-
***Ligilactobacillus***	*L. murinus*	NM26	**+**
		NM28	**+**
	*L. ruminis*	DSM20403	**+**
***Lactobacillus***	*L. johnsonii*	NM60	**+**
	*L. intestinalis*	NM61	**+**
	*L. gasseri*	JV-V03	**+**
		EX336960VC01	**+**
	*L. crispatus*	PSS7772C	**+**
		EX849587VC03	**+**
	*L. jensenii*	269–3	**+**
		EX849587VC03	**+**
	*L. iners*	SPIN 2503V10-D	**+**
	*L. psittaci*	DSM15354	**+**
	*L. delbrueckii*	N/A	**+**
***Lacticaseibacillus***	*L. rhamnosus*	LMS2-1	**+**
***Lactiplantibacillus***	*L. plantarum*	DSM20174	**+**
***Loigolactobacillus***	*L. coryniformis*	DSM20001	**+**
***Companilactobacillus***	*C. farciminis*	DSM20184	**+**
	*C. alimentarius*	DSM20249	**+**
***Schleiferilactobacillus***	*S. shenzhenensis*	DSM28193	**+**

**Figure 2.**
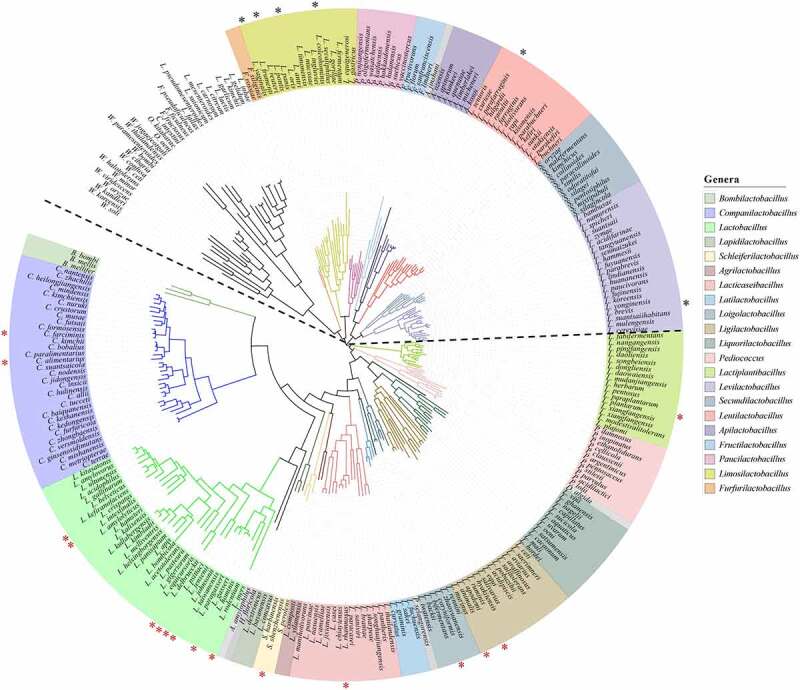
Inhibitory and uninhibitory *Lactobacillaceae* species cluster according to phylogeny. Phylogenomic analysis and tree constructed based on the concatenated alignment of protein sequences for 114 single-copy genes from type strains, containing 244 species from the *Lactobacillaceae* family (Zheng et al., 2020). Newly created genera (from what was previously classified as the *Lactobacillus* genus) are indicated by branch and label colors, and the legend on the right shows the name of each genus. Species labeled in gray are the sole representatives from their genus. Unlabeled species belong to the closely related *Leuconostocaceae* family (recently amalgamated into the *Lactobacillaceae* family). Species selected and tested against representatives from S24-7 (NM74_B14) and *Lachnospiraceae* (NM01_1-7b) are indicated by asterisks. Species that inhibited growth are below the dotted line, indicated by red asterisks. Species that were uninhibitory are above the dotted line, indicated by black asterisks.

Strikingly, the inhibitory genera clustered together both phylogenetically and according to their primary mode of fermentation (Figure 2). The inhibitory isolates all belonged to the homofermentative group of lactic acid bacteria that primarily use the Embden-Meyerhof pathway to metabolize hexoses, whereas the uninhibitory isolates were all heterofermentative bacteria that utilize the phosphoketolase pathway. The genomes of most isolates in our *Lactobacillaceae* collection were available and their putative metabolic capacities were further assessed using the KofamScan gene function annotation tool provided by the Kyoto Encyclopedia of Genes and Genomes (KEGG). This analysis revealed that all inhibitory strains encoded either 6-phosphofructokinase (*pfkA*, KEGG Orthology Number K00850) or 1-phosphofructokinase (*fruK*, K00882), while all uninhibitory strains lacked these genes (Table S3). Both phosphofructokinases generate fructose-1,6-bisphosphate, a central metabolic intermediate of the Embden-Meyerhof pathway, and their presence/absence is used to differentiate homofermentative from heterofermentative *Lactobacillaceae*.^10,30^ Additionally, two genes from the phosphoenolpyruvate-dependent sugar phosphotransferase system (PTS), *fruA* (K02770, transports fructose), and *manX* (K02794, transports mannose), were uniformly present in inhibitory strains and absent from uninhibitory strains. This coincides with the observation that heterofermentative *Lactobacillaceae* species harbor fewer PTS than homofermentative species, which is associated with a general loss of gene families related to carbohydrate transport and metabolism.^10^ The only KEGG functions identified that were common to uninhibitory strains and absent from all uninhibitory strains were 1,3-propanediol dehydrogenase (*dhaT*, K00086), which enables homofermentative lactobacilli to utilize glycerol as a hydrogen acceptor during the fermentation of glucose, and an uncharacterized putative glyoxalase (*phnB*, K04750).^31,32^

### *Inhibitory* Lactobacillaceae *produce considerably higher quantities of lactate and pyruvate and lower the pH of the surrounding media*

To define the metabolites produced by uninhibitory *L. reuteri* and *L. brevis*, and inhibitory *L. murinus* and *L. plantarum*, a quantitative compositional analysis of culture supernatants was performed using anion chromatography. Although the levels of several metabolites varied across each species, most did not correlate with the inhibitory and uninhibitory groups. However, the concentrations of lactate and formate were on average 1.5× and 2.0× higher, respectively, in inhibitory *L. murinus* and *L. plantarum* supernatants compared to uninhibitory *L. reuteri* and *L. brevis* supernatants (Table 2). The concentration of pyruvate was also elevated for inhibitory supernatants, with *L. murinus* containing 6.8 mg/L and *L. plantarum* containing 1.6 mg/L compared to 0.4 mg/L for *L. reuteri* and 0.1 mg/L for *L. brevis*. The higher concentrations of lactate and pyruvate in *L. murinus* and *L. plantarum* supernatant is consistent with their use of homofermentative metabolism, as lactate is the main end-product of this process and pyruvate is a key intermediate metabolite. Increased formate production by *L. murinus* and *L. plantarum* is likely due to the presence of formate C-acetyltransferase (*pflB*) in both genomes, a gene encoding an enzyme which catalyzes the conversion of pyruvate and coenzyme A (CoA) into formate and acetyl-CoA (Table S3). *L. reuteri* and *L. brevis* as well as other uninhibitory species lack this gene, however *pflB* is not found in all inhibitory strains of lactobacilli and therefore formate production is unlikely to be a central player in the inhibition of S24-7 and *Lachnospiraceae*.

**Table 2.** Quantitative chromatographic analysis of anions present in culture supernatant of heterofermentative (inhibitory) and homofermentative (uninhibitory) *Lactobacillaceae* strains. Values are the average of three independent experiments. Standard deviation is shown for each value. n.d. = not detected

			Heterofermentative (uninhibitory)		Homofermentative (inhibitory)	
Anions		Blank media (mg/L)	*L. reuteri* NM11 (mg/L)	*L. brevis* (mg/L)	*L. murinus* NM26 (mg/L)	*L. plantarum* (mg/L)
**Organic**	**Lactate**	1.113 ± 0.050	14.914 ± 0.105	14.479 ± 0.176	21.387 ± 0.472	23.135 ± 0.244
	**Acetate**	2.661 ± 0.180	5.039 ± 0.051	5.726 ± 0.255	2.972 ± 0.276	5.973 ± 0.074
	**Propionate**	0.018 ± 0.015	n.d.	n.d.	n.d.	n.d.
	**Formate**	1.206 ± 0.077	1.110 ± 0.007	1.132 ± 0.014	2.124 ± 0.076	2.337 ± 0.031
	**Butyrate**	0.016 ± 0.003	0.072 ± 0.001	0.098 ± 0.001	0.042 ± 0.001	0.090 ± 0.015
	**Pyruvate**	9.362 ± 0.609	0.405 ± 0.048	0.118 ± 0.001	6.780 ± 0.224	1.595 ± 0.123
	**Succinate/Malate**	0.454 ± 0.031	0.427 ± 0.010	0.442 ± 0.003	0.442 ± 0.010	1.095 ± 0.013
	**Fumarate**	0.612 ± 0.081	0.444 ± 0.006	0.462 ± 0.014	0.699 ± 0.027	0.489 ± 0.076
	**Citrate**	0.812 ± 0.090	0.804 ± 0.004	0.809 ± 0.007	0.785 ± 0.022	0.023 ± 0.006
**Inorganic**	**Chloride**	103.504 ± 6.783	98.583 ± 0.401	99.011 ± 0.972	99.002 ± 2.222	99.219 ± 1.254
	**Nitrite**	0.002 ± 0.003	n.d.	n.d.	n.d.	n.d.
	**Sulfate**	0.075 ± 0.004	0.141 ± 0.004	0.079 ± 0.015	0.082 ± 0.004	0.115 ± 0.003
	**Nitrate**	0.825 ± 0.058	0.812 ± 0.005	0.796 ± 0.008	0.801 ± 0.020	0.820 ± 0.019
	**Phosphate**	16.718 ± 1.076	15.718 ± 0.033	15.794 ± 0.201	15.598 ± 0.375	15.284 ± 0.130

**Figure 3.**
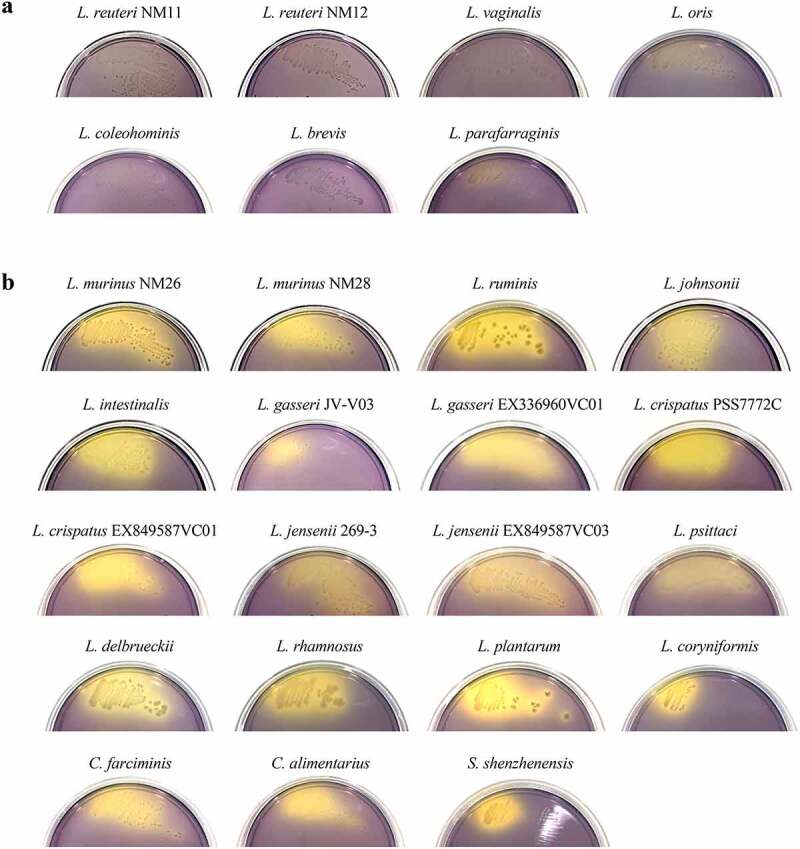
Inhibitory *Lactobacillaceae* species produce more acid than uninhibitory species. Each *Lactobacillaceae* strain was streaked out onto plates containing the pH indicator bromocresol purple (purple above pH 6.8, yellow below pH 5.2). Representative images are shown (n = 3). A) Strains that showed no signs of inhibition of NM74_B14 (S24-7) or NM01_1-7b (*Lachnospiraceae*). B) Strains that inhibited growth of both NM74_B14 and NM01_1-7b. *L. iners* was excluded as it did not grow on these plates.

An increase in acid production by homofermentative species might be expected to impact the pH of the culture media to a greater extent than for heterofermentative species. To determine whether homofermentative *Lactobacillaceae* have a pronounced effect on local pH, we assessed the effect of each strain on agar plates using the pH indicator bromocresol purple. Although all heterofermentative (uninhibitory) strains grew robustly on these plates, each one only produced a minimal shift in the pH of the surrounding media (Figure 3a). Conversely, all of the homofermentative (inhibitory) strains caused extensive acidification of the agar to below the pKa of 6.3 of bromocresol purple (Figure 3b). This strong effect on pH was notable even for the isolates that grew poorly on this media, such as *L. gasseri* JV-V03, *L. coryniformis*, and *S. shenzhenensis*. This demonstrated a clear correlation between media acidification and the ability of specific lactobacilli to inhibit the growth of S24-7 and *Lachnospiraceae* isolates.

### *Mitigating acidity alleviates the inhibitory effect of* Lactobacillaceae *species*

**Figure 4.**
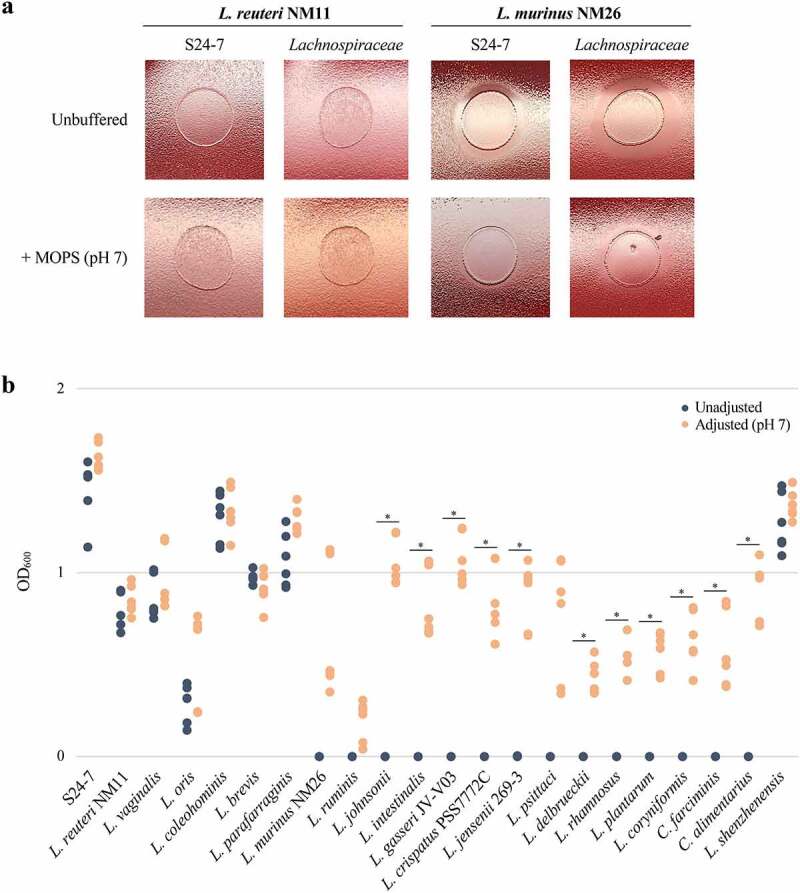
Mitigating acidity alleviates the inhibitory effect of *Lactobacillaceae* species against both S24-7 and *Lachnospiraceae*. A) Uninhibitory *L. reuteri* NM11 and inhibitory *L. murinus* NM26 spotted onto lawns of NM74_B14 (S24-7) and NM01_1-7b (*Lachnospiraceae*). The top row of plates is unbuffered, while the bottom row of plates are supplemented with MOPS buffer at pH 7. Representative images are shown (n = 3). B) Growth measured by OD_600_ of NM74_B14 in supernatant from liquid cultures of uninhibitory and inhibitory *Lactobacillaceae* species. For each *Lactobacillaceae* species, blue data points show growth of NM74_B14 in unadjusted supernatant while orange data points show growth in supernatant adjusted to pH 7 using NaOH (as indicated in the legend on the upper right). *L. iners* was excluded as it did not grow in this liquid media. ‘S24-7’ is a control of NM74_B14 grown in its own supernatant. Data points are from three independent experiments. Welch’s t-test, * = p < .05.

To test if acidification of the surrounding media is the underlying cause of growth inhibition, *L. reuteri* (uninhibitory) and *L. murinus* (inhibitory) were spotted onto lawns of NM74_B14 or NM01_1-7b, either on standard media or media supplemented with MOPS buffer adjusted to pH 7. MOPS buffer had no effect on the growth of either NM74_B14 or NM01_1-7b when grown with *L. reuteri*, while the zones of growth inhibition for both isolates when grown with *L. murinus* were markedly reduced when the agar was supplemented with MOPS (Figure 4a).

To examine the influence of pH more extensively across our entire collection of *Lactobacillaceae* species, we selected one isolate of each species to test the effects of their respective culture supernatants on the growth of the S24-7 strain NM74_B14 (with the exception of *L. iners*, which was unable to grow under these conditions). Supernatants were taken from cultures of each *Lactobacillaceae* species, either adjusted to pH 7 or left unadjusted, and subsequently inoculated with NM74_B14 to evaluate growth. Both turbidity and pH were measured for each *Lactobacillaceae* culture before taking supernatant, and turbidity measurements varied dramatically between *Lactobacillaceae* species independent of inhibitory capacity (Table S4). The pH of uninhibitory cultures ranged from pH 6.3–6.7 compared to pH 5.2–6.3 for inhibitory cultures, thereby corroborating the observations from the bromocresol purple assay. In the supernatants of uninhibitory *Lactobacillaceae* species NM74_B14 grew with and without pH adjustment, while for inhibitory species it only grew when the pH was adjusted to 7.0 (Figure 4b). The exception was *S. shenzhenensis* (inhibitory), which grew poorly in the liquid media we used and maintained a high culture pH relative to other inhibitory isolates. NM74_B14 grew in both unadjusted and adjusted *S. shenzhenensis* supernatants.

Due to the extremely poor growth of *Lachnospiraceae* isolates in liquid media, this assay could not be performed with NM01_1-7b. Instead, spotting assays were carried out on agar plates using MOPS to help buffer pH. In the presence of heterofermentative (uninhibitory) *Lactobacillaceae*, growth of NM01_1-7b was robust on both MOPS-supplemented and unbuffered media. In contrast, the zones of growth inhibition surrounding culture spots of homofermentative (inhibitory) *Lactobacillaceae* were substantially reduced on plates supplemented with MOPS buffer (Figure S5). Together, these findings indicate that media acidification is required for the inhibitory activity of homofermentative *Lactobacillaceae* against S24-7 and *Lachnospiraceae*.

### *S24s-7 and* Lachnospiraceae *species are more acid-sensitive than related species within the* Bacteroidales *and* Clostridiales *orders*

Our initial screen examining the effect of lactobacilli on the growth of other members of the CIAMIB collection suggested that acid sensitivity might be characteristic of S24-7 and *Lachnospiraceae*, but not other *Bacteroidales* or *Clostridiales* bacteria. To investigate this, we selected species from the CIAMIB that belong to the *Bacteroidales* and *Clostridiales* orders most closely related to S24-7 and *Lachnospiraceae* species, respectively (Figure S6). *Phocaeicola sartorii, Parabacteroides distasonis, Bacteroides faecichinchillae*, and *Bacteroides caecimuris* were chosen as representatives from the *Bacteroidaceae* family within the *Bacteroidales* order, while *Clostridium perfringens, Clostridium sartagoforme*, and *Clostridium chromiireducens* were selected from the *Clostridiaceae* family within the *Clostridiales* order (Table S1).

**Figure 5.**
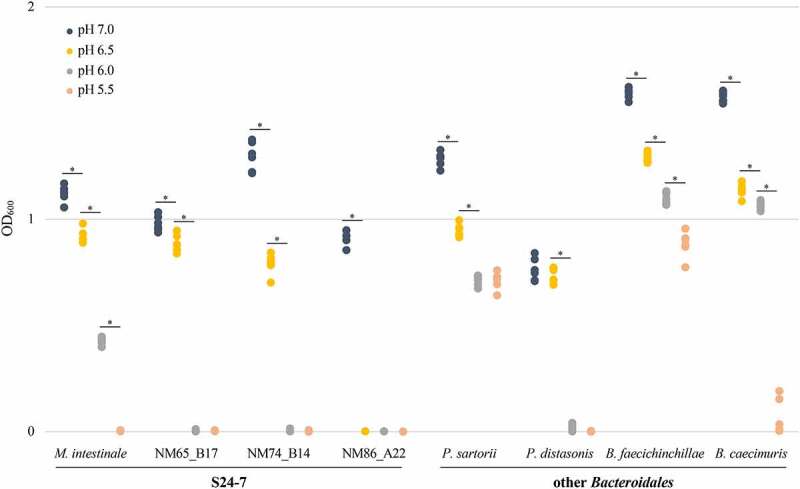
S24-7 species are more sensitive to acidity than other species within the *Bacteroidales* order. The four S24-7 isolates from the CIAMIB and four related species from within the *Bacteroidales* order were cultured in liquid media at pH 7.0, 6.5, 6.0, and 5.5, as indicated by the legend on the upper left. For each species at each pH, OD_600_ was measured 72 hours after inoculation as a proxy for growth. Data points are from three independent experiments. Welch’s t-test, * = p < .05.

The growth of S24-7 isolates *M. intestinale*, NM65_B17, NM74_B14, and NM86_A22 was compared against the four *Bacteroidaceae* species in liquid media adjusted to several pH levels (Figure 5). Despite variation in the extent of growth, at pH 7 both S24-7 and *Bacteroidaceae* species grew robustly. However, each decrease in pH considerably reduced growth of S24-7 species, and by pH 5.5 none showed any growth. Contrarily, *P. sartorii, B. faecichinchillae*, and *B. caecimuris* continued to grow even at pH 5.5. *P. distasonis* stopped growing at pH 6.0, but unlike S24-7 species its growth was not significantly reduced at pH 6.5 compared to pH 7.0. This increased sensitivity of *P. distasonis* at lower pH levels compared to other *Bacteroidaceae* species may reflect the phylogeny of these strains, as *P. distasonis* clusters more closely with the S24-7 species than the *Bacteroidaceae* species (Figure S6).

**Figure 6.**
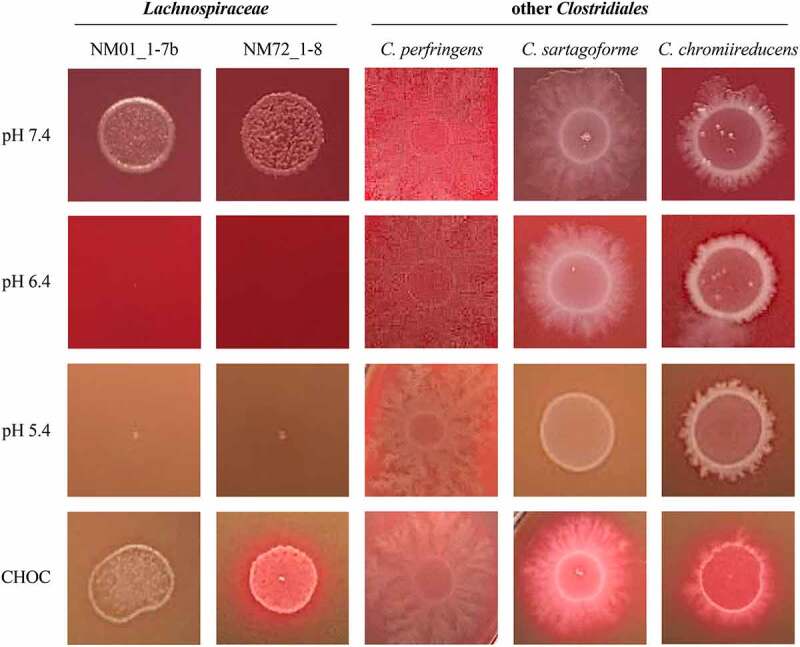
*Lachnospiraceae* species are more sensitive to acidity than other species within the *Clostridiales* order. The two *Lachnospiraceae* isolates from the CIAMIB and three related species from within the *Clostridiales* order were spotted onto agar plates adjusted to pH 7.4, 6.4, and 5.4, as well as chocolate agar (CHOC) plates. Representative images are shown (n = 3).

The growth of *Lachnospiraceae* isolates NM01_1-7b and NM72_1-8 was compared against the three *Clostridiaceae* species by spotting onto plates adjusted to several pH levels (Figure 6). On plates where pH had not been adjusted (pH 7.4), both *Lachnospiraceae* and *Clostridiaceae* species grew robustly. On plates adjusted to pH 6.4 and 5.4, growth of both *Lachnospiraceae* isolates was completely inhibited whereas all *Clostridiaceae* species continued to grow well. For these assays we employed agar containing red blood cells and noticed that lysis of these cells was extensive on plates adjusted to pH 5.4. To control for the possibility that blood cell lysis released compounds that inhibit *Lachnospiraceae* independent of pH we examined the growth of *Lachnospiraceae* on chocolate agar plates, which are formulated using lysed red blood cells. All species grew on chocolate agar plates at neutral pH. Collectively, these solid and liquid growth assays established that species belonging to the S24-7 group and the *Lachnospiraceae* family are highly susceptible to acidic conditions, a feature they do not share with related taxa abundant in the gut microbiota.

## Discussion

Here, we report the inhibition of representatives from the prevalent *Lachnospiraceae* family and S24-7 group by a diverse set of *Lactobacillaceae* species. We find that this inhibitory activity is mediated by increased acidity and that it is congruent with the current phylogenetic and metabolic categorizations of the *Lactobacillaceae* family. This represents one of the first in-depth explorations into the effect of a broad range of *Lactobacillaceae* species on distinct taxa from the commensal gut microbiota. It is also the first report, to our knowledge, that *Lachnospiraceae* and S24-7 bacteria are highly sensitive to acid stress.

The benefits that *Lactobacillaceae* species have on human and animal health are frequently emphasized. However, many studies regarding the probiotic properties of lactobacilli do not address the physiological or ecological characteristics of the species or strain being studied and rarely is the impact these bacteria might have on the larger gut microbial ecosystem considered. The recent re-classification of fermentation types and division of this genus into multiple genera based on phylogeny, physiology, and ecology is a major step toward elucidating the hallmarks of each clade within the *Lactobacillaceae* family.^6,10^ These updated designations allowed us to determine that only homofermentative *Lactobacillaceae* species exert an inhibitory effect on *Lachnospiraceae* and S24-7 species.

Organic acid production and its effect on local pH levels has not been systematically explored as it relates to homofermentative and heterofermentative lactobacilli, nor have the ecological consequences of these differences. Prior work has demonstrated that homofermentative species produce high levels of lactic acid while heterofermentative species produce a mix of lactic acid and acetic acid.^33–36^ We found that homofermentative strains generated almost half again as much lactate as heterofermentative strains. Formate production was also considerably higher in homofermentative strains, although this is likely due to the presence of formate C-acetyltransferase in the genomes of both the *L. murinus* and *L. plantarum* isolates we selected to study. Conversely, acetate levels were comparable across groups.

The increased acidity that we observed in the media of homofermentative species may be a direct consequence of producing more organic acid, but the differences in total acid production were not as extreme as the differences in culture pH would indicate. It is possible that heterofermentative species buffer their surrounding environment through the production of ammonia or proton consumption via enzymes such as urease, arginine deiminase, and glutamate decarboxylase.^37–41^ However, the simplistic explanation that heterofermentative species actively neutralize their surrounding environment while homofermentative strains do not is complicated by the fact that many homofermentative species also encode deaminase and decarboxylase enzymes in their genomes.

The capacity of organic acids to inhibit bacterial growth is well established.^14,18,19,42^ One question is whether pH is the direct cause of growth inhibition as opposed to a specific organic acid such as lactate, but our experiments suggest that low pH is sufficient to inhibit *Lachnospiraceae* and S24-7 growth. We note that S24-7 was able to grow in *Lactobacillaceae* supernatants adjusted to a neutral pH, hence neutral lactate and acetate were not inhibitory. Additionally, neither S24-7 nor *Lachnospiraceae* were able to grow on media where the pH was lowered using inorganic acid – although the media was complex and may have contained low amounts of lactate and other acid salts.

The *Lachnospiraceae* and S24-7 species are poorly studied, largely due to a paucity of cultured isolates, and their sensitivity to stress has not been extensively examined. The apparent acid sensitivity of both taxa relative to other species from the *Bacteroidales* and *Clostridiales* orders warrants further investigation. The S24-7 group of bacteria has been shown to be highly intolerant to hyperosmotic conditions.^43^ Indeed, osmotic-induced diarrhea caused by the administration of polyethylene glycol (as routinely occurs prior to colonoscopy procedures) leads to an extinction of these bacteria from the gut. An inability to survive acid stress could render both S24-7 and *Lachnospiraceae* susceptible to elimination from the gut under circumstances that lower the pH of the colon including chronic inflammation or certain drugs, food additives, or xenobiotics.

It remains an open question whether interactions between lactobacilli and S24-7 or *Lachnospiraceae* occur in nature and are relevant in the context of the gut. *Lachnospiraceae* and S24-7 species are found in the colon (and cecum in mice) where the pH is typically close to neutral. *Lactobacillaceae* species, in contrast, are usually more abundant in the small intestine and stomach, where the pH is considerably more acidic.^44–47^ The host and other bacteria also modulate the composition and presence of organic acids through various mechanisms. A number of commensal gut bacteria from the *Firmicutes* phylum are able to convert lactate and acetate to short-chain fatty acids, particularly butyrate.^48–50^ In turn, the intestinal mucosa of the host can absorb lactate and short-chain fatty acids and control the pH of small intestinal contents through secretion of bicarbonate.^51–54^

There are many scenarios where inhibitory activity of specific lactobacilli on the gut microbiota could be important, including during the administration of lactobacilli as probiotics. *Lactobacillaceae* strains such as *L. rhamnosus* GG have been shown to at least transiently colonize the human colonic mucosa.^55,56^ Furthermore, the prevalence of both *Lachnospiraceae* and S24-7 species has been altered in numerous studies examining the effect of *Lactobacillaceae* species in food and probiotics on the gut microbiota.^22,57–63^ The effect that lactobacilli have on commensal bacteria like S24-7 and *Lachnospiraceae* may explain why administering some probiotics delays the reestablishment of the endogenous microbiota after antibiotic treatment. Regardless, this work highlights both the sensitivity of two abundant members of the gut microbiota to stress and the need to better characterize the effects that different species (and strains) of probiotic bacteria can have on the gut microbiota as a whole.

## Methods

### Bacterial growth media and conditions

All bacterial growth was carried out at 37°C in an anaerobic chamber (Anaerobe Systems, AS-580) using a gas mixture of 10% hydrogen, 10% carbon dioxide, and 80% nitrogen. Culture media was always transferred to anaerobic conditions at least 24 hours before use. Brucella Blood Agar (BRU) supplemented with hemin and vitamin K (Hardy Diagnostics, A30) was used to grow bacterial strains on solid media unless otherwise specified. For all assays, bacteria were streaked out onto BRU plates from stocks stored at −80°C in 10% glycerol and grown for 48–72 hours, then re-streaked onto fresh BRU plates and grown for another 48–72 hours. Brain Heart Infusion (BHI) Broth (Oxoid, CM1135) supplemented with 5 μg/mL hemin (Sigma, 51280), 1 μg/mL vitamin K_1_ (Sigma, 95271), and 0.5 mg/mL L-cysteine HCl after autoclaving was used to grow bacterial strains in liquid media. For liquid assays not requiring co-culturing or subsequent growth of NM74_B14 or NM01_1-7b, *Lactobacillaceae* species were cultured in de Man, Rogosa, and Sharpe (MRS) Broth (Sigma-Aldrich, 69966).

### Spotting assays

For initial spotting assays using *Lactobacillaceae* species from the CIAMIB, S24-7 and *Lachnospiraceae* species were resuspended directly from BRU plates in BHI. S24-7 species were diluted to an OD_600_ of 0.1 and *Lachnospiraceae* species were diluted to an OD_600_ of 0.6 (due to their poor growth). For all subsequent spotting assays, a 1 μL inoculation loop full of NM74_B14 or NM01_1-7b from BRU plates was added to 4 mL of BHI to propagate enough bacteria to enable spotting of all *Lactobacillaceae* species. Cultures were incubated for 24 hours then diluted to an OD_600_ of 0.1 or 0.6, respectively. From this point onward, all spotting assays followed the same procedure. To create bacterial lawns, 600 μL of each dilution was spread onto individual BRU plates. Plates were tilted to evenly distribute bacteria, as using a spreader produced uneven swathes of S24-7 and *Lachnospiraceae* growth. Each *Lactobacillaceae* species was resuspended in 1X phosphate-buffered saline (PBS) containing 0.1% L-cysteine HCl and diluted to an OD_600_ of 1. After S24-7 or *Lachnospiraceae* lawns had dried, 5 μL of each *Lactobacillaceae* strain was spotted on top. Plates were incubated for 72 hours and imaged.

To test the effect of *Lactobacillaceae* supernatant and heat-killed bacteria compared to live bacteria, liquid cultures of NM74_B14 and NM01_1-7b (as described above) were grown for 24 hours, diluted to an OD_600_ of 1 and filtered using a 0.22 μM filter (to obtain supernatant), boiled for 30 minutes (to heat-kill bacteria), or left unmodified (live bacteria). For each *Lactobacillaceae* species tested, separate tubes containing 3.6 mL of MRS were supplemented with 400 μL of supernatant, heat-killed, or live NM74_B14 or NM01_1-7b. Each of these mixtures was inoculated with individual *Lactobacillaceae* species from BRU plates diluted as described above, alongside a set of tubes containing 4 mL MRS. After incubating for 24 hours, supernatant and heat-killed bacteria were obtained from each of these cultures by filtering and boiling. These combinations, as well as live bacteria, were then spotted onto lawns of NM74_B14 or NM01_1-7b in the same manner as described for previous spotting assays. Plates were incubated for 72 hours and imaged.

For buffered media assays, 1 M MOPS was prepared by dissolving 10.46 g MOPS (BioShop, MOP001) in distilled water (dH_2_O), adjusting to pH 7 with 10 N NaOH, filling to 50 mL, and filter-sterilizing. 200 μL of this 1 M MOPS buffered at pH 7 was spread onto BRU plates and allowed to dry completely before proceeding with spreading lawns of NM74_B14 and NM01_1-7b and spotting as described previously. Plates were incubated for 72 hours and imaged.

### Co-culturing assay

NM74_B14 and *Lactobacillaceae* strains *L. reuteri* NM11 and *L. murinus* NM26 were each resuspended in BHI and diluted to an OD_600_ of 4. For axenic cultures, 5 μL of diluted NM74_B14, *L. reuteri* NM11, or *L. murinus* NM26 was added to 4 mL of BHI. For co-cultures, 5 μL of *L. murinus* NM11 or *L. murinus* NM26 was added along with 5 μL of NM74_B14 to 4 mL of BHI. Cultures were incubated for 72 hours, at which point OD_600_ was measured.

For quantitative PCR (qPCR), individual reactions were set up with 10 μL SsoFast EvaGreen Supermix (Bio-Rad, 1725203), 1 μL each of 10 μM forward and reverse primers, and 8 μL template DNA diluted 10X in nuclease-free water, to a total reaction volume of 20 μL. Primers for *L. reuteri* NM11 (forward: 5’-GGACTACCAGGGTATCTAA-3’; reverse: 5’-TCTCAACACCCGCCTTAATC-3’), *L. murinus* NM26 (forward: 5’-CCACATGCTAGTGAGCGTATC-3’; reverse: 5’-GTCCAGTTTCTTCTCGCTTCT-3’), and NM74_B14 (forward: 5’-GTGGAAACGAGAAGACTGTAGAA-3’; reverse: 5’-TTTCGTCTCTCAATCGGGAATAG-3’) were designed for this study. These primer sets targeted genes unique to each species (hypothetical proteins for *L. reuteri* and *L. murinus*, and a FMN adenylyltransferase/riboflavin kinase for NM74_B14) to ensure specificity, which was checked using PrimerBlast and confirmed experimentally. qPCR was carried out on an Eppendorf Mastercycler ep realplex in a 96-well format. Cycling conditions were 30 seconds at 95°C, 40 cycles of 5 seconds at 95°C and 10 seconds at 60°C, 15 seconds at 95°C followed by 15 seconds at 60°C, and a 20-minute ramp up to 95°C for 15 seconds. Five-point standard curves of 10X dilutions (20 ng to 0.002 ng or 10 ng to 0.001 ng) were set up in duplicate, while each sample was run in triplicate. Analysis of qPCR efficiency and accuracy was carried out using Eppendorf realplex software.

### Transwell plate assay

NM74_B14 and *Lactobacillaceae* strains *L. reuteri* NM11 and *L. murinus* NM26 were resuspended from BRU plates in BHI. NM74_B14 was diluted to an OD_600_ of 0.6 and *Lactobacillaceae* strains were diluted to an OD_600_ of 0.1. 600 μL of BHI was aliquoted into the bottom compartment of the transwell plate (VWR, 10769–198) and 100 μL was aliquoted into the insert, which contains a 0.1 μM filter. Next, 5 μL of NM74_B14 was added to the bottom compartment and 5 μL of *L. reuteri* NM11 and *L. murinus* NM26 was added to separate inserts. Cultures were incubated for 72 hours, at which point OD_600_ was measured.

### Phylogenetic tree construction

For the *Lactobacillaceae* family, phylogenomic analysis and tree were generated as described in Zheng et al., based on the concatenated alignment of protein sequences for 114 single-copy genes from type strains of all available *Lactobacillaceae* and *Leuconostocaceae* species retrieved August 2019 from GenBank.^6^ Visualization was performed with the Interactive Tree of Life.^64^ For clarity, both branches and bacterial names were highlighted based on the new *Lactobacillaceae* genera assignments, and a corresponding legend was added to allow easy referencing of updated groupings and genus names.

Phylogenetic trees of S24-7 and *Bacteroidaceae* species, as well as *Lachnospiraceae* and *Clostridiaceae* species, were constructed using 16S rRNA gene sequences. The full length (>1400 nucleotides) sequences of the 16S rRNA genes for each species were aligned with MUSCLE in MEGA7 using a 97% cutoff value.^65,66^ *Psychroflexus gondwanensis* (16S rRNA gene accession: JX986967.1) was used as an outgroup for the *Bacteroidales* tree. NM09_H32 from the CIAMIB (16S rRNA gene accession: MK929057.1) was used as an outgroup for the *Clostridiales* tree. This alignment was used to construct phylogenetic trees with the Neighbor-Joining method in MEGA7, with a bootstrap replication of 1000.^66,67^ Modifications to annotations were carried out in Inkscape 1.1.

### Genomic analyses

Genomic comparisons between *Lactobacillaceae* species from the CIAMIB were conducted on the Pathosystems Resource Integration Center (PATRIC) through their Bacterial Bioinformatics Resource Center, using the genome assemblies for *L. reuteri* NM11, *L. murinus* NM26, *L. johnsonii*, and *L. intestinalis* (provided in Table S1).^68^ Bidirectional BLASTP was used to perform protein sequence-based genome comparisons in a pairwise manner between each species. The following default parameters were used: 30% minimum coverage of query and subject in BLAST; maximum E value of 1e-5; and 10% minimum identity of query and subject in BLAST.

For comparing KEGG Orthologs (KOs) between *Lactobacillaceae* species, coding sequences from the genome assemblies of all strains were downloaded from the National Center for Biotechnology Information (NCBI) RefSeq database in FASTA Nucleotide format. The Bio.SeqIO package from Biopython was used to translate each coding sequence to generate protein FASTA files for each genome.^69^ KofamScan version 1.3.0 was used to search each translated coding sequence against the KEGG database of Hidden Markov Models (HMMs), which represents a broad range of defined protein families.^70^ Significant hits were identified as those scoring above HMM-specific thresholds, and the KO identifier for the corresponding family was assigned to each translated coding sequence. All *Lactobacillaceae* strains in our collection were included in this analysis, with the exception of *L. vaginalis, L. gasseri* EX336960VC01, *L. crispatus* EX849587VC01, *L. jensenii* EX849587VC03, and *L. delbrueckii* as sequences for these strains were not available.

### Anion chromatography

Each *Lactobacillaceae* strain was resuspended from BRU plates in BHI and diluted to an OD_600_ of 4, then 5 μL of this resuspension was transferred to 4 mL of BHI. Cultures were incubated for 24 hours, at which point supernatants were obtained for each strain by filtering cultures through 0.22 μM filters. Samples were frozen at −80°C until metabolic profiling was conducted. After thawing in preparation for chromatography, samples were diluted 20× and standards for anions of interest (acetate, chloride, citrate, citrate, formate, fumarate, lactate, nitrate, phosphate, pyruvate/oxaloacetate, and succinate/malate) were run at the following concentrations to generate standard curves: 0.5 mM, 0.2 mM, 0.05 mM, 0.01 mM, and 0.005 mM. Anion chromatography was carried out by the BioZone facility at the University of Toronto on a Thermo Scientific Dionex ICS-2100 ion chromatography system using an IonPac AS11 IC Column. For both standards and samples, the injection volume onto the column was 20 μL and the run time was 45 minutes. The flow rate was set to 1.0 mL/minute and the suppressor was set at 30 mM maximum [OH^−^] with a current of 75 mA. The eluent was a multi-step gradient of KOH that started at 0.5 mM. Data analysis was conducted on the Chromeleon Chromatography Data System software.

### pH indicator plates

Indicator plates were created by supplementing 1 L worth of BHI with 15 g of agar and 0.02 g of bromocresol purple before autoclaving. After autoclaving, the agar was supplemented with 5 μg/mL hemin, 1 μg/mL vitamin K_1_, and 0.5 mg/mL L-cysteine HCl. *Lactobacillaceae* strains grown on BRU plates were resuspended in 1X PBS and diluted to an OD_600_ of 1. A 1 μL inoculation loop full of each strain was streaked out onto bromocresol purple agar plates. Plates were incubated for 72 hours, at which point images were taken.

### Supernatant assay

*Lactobacillaceae* strains and NM74_B14 were individually resuspended from BRU plates in BHI and diluted to an OD_600_ of 4, then 13.75 μL of this resuspension was transferred to 11 mL of BHI. Cultures were incubated for 24 hours, then filtered with 0.22 μM filters to obtain 10 mL of supernatant. To adjust pH, a 2 mL aliquot of each supernatant was measured and adjusted to pH 7 using 10 N NaOH. The remaining 8 mL of supernatant was split into four 2 mL aliquots, and two of these aliquots were adjusted to pH 7 using the same quantity of 10 N NaOH (without measuring pH, to maintain sterility). Next, NM74_B14 was resuspended from BRU plates into BHI and diluted to an OD_600_ of 2. 5 μL of this dilution was added to each *Lactobacillaceae* supernatant and the NM74_B14 supernatant as a control. Cultures were incubated for 72 hours, at which point OD_600_ was measured.

### Testing acid sensitivity

For liquid assays with S24-7 and other *Bacteroidales* species, BHI was adjusted to pH 7.0, 6.5, 6.0, and 5.5 using 6 N HCl. After adjusting pH, media was filter-sterilized through a 0.22 μM filter and added in 2 mL aliquots to individual wells in 24-well plates. Each *Bacteroidales* species was resuspended in BHI from BRU plates and diluted to an OD_600_ of 2, then 5 μL of each species was added to aliquots of media adjusted to the four different pH levels. Cultures were incubated for 72 hours, at which point OD_600_ was measured.

For plate assays with *Lachnospiraceae* and other *Clostridiales* species, customized plates were created using Brucella Broth (BD, B11088) with 15 g of agar per 1 L of dH_2_O. After autoclaving, media was supplemented with 50 mL of sheep blood (Cedarlane, CL2581-100D). At this point, media was left either unmodified (pH 7.4), or adjusted to pH 6.4 or 5.4 by adding 6 N HCl. To make chocolate agar (CHOC), sheep blood was warmed in a water bath to 55°C for two hours before adding to media. Cultures of *Clostridiales* species were set up by taking a 1 μL inoculation loop full of each species and inoculating 4 mL of BHI. After incubating for 24 hours, cultures were diluted to an OD_600_ of 0.6 and 10 μL of each species was spotted onto pH-adjusted and CHOC plates. Plates were incubated for 72 hours, at which point images were taken.

## Supplementary Material

Supplemental Material

## Data Availability

The data that support the findings of this study are available from the corresponding author (WWN) upon reasonable request. Genomic sequence data from previously unpublished strains that appear in this manuscript can be obtained from NCBI Bioproject #474907. Strains used in this study are available from WWN.
