# Supplementary material for: Acids produced by lactobacilli inhibit the growth of commensal Lachnospiraceae and S24-7 bacteria
Source: Gut Microbes. 2022 Mar 10;14(1):2046452. doi: 10.1080/19490976.2022.2046452 (PMC8920129; doi:10.1080/19490976.2022.2046452)
Supplement: Supplemental Material [file KGMI_A_2046452_SM4942.zip › 2.pdf]

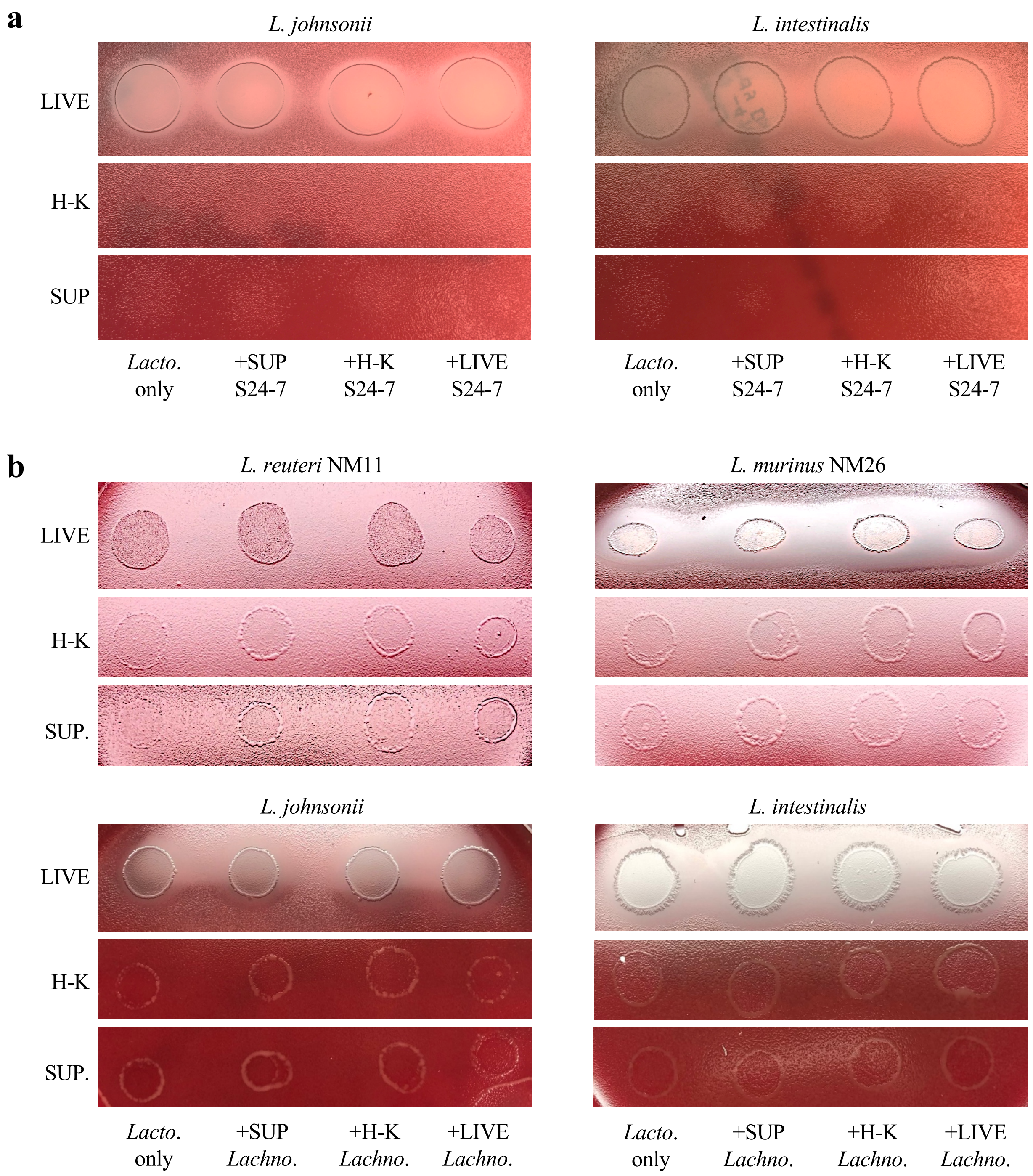

Supplementary Figure 2. Effect of supernatant, heat-killed, and live *Lactobacillaceae* species on growth of S24-7 and *Lachnospiraceae* species. Representative images are shown (n = 3). (a) Live (LIVE), heat-killed (H-K), and supernatant (SUP) of *L. johnsonii* and *L. intestinalis* liquid cultures spotted onto a lawn of NM74_B14 (S24-7). From left to right, spotting is *Lactobacillaceae* grown in liquid media alone (*Lacto*. only), with NM74_B14 supernatant (+SUP S24-7), heat-killed (+H-K S24-7), and live (+LIVE S24-7). (b) Live (LIVE), heat-killed (H-K), and supernatant (SUP) of *L. reuteri* NM11, *L. murinus* NM26, *L. johnsonii*, and *L. intestinalis* spotted onto a lawn of NM01_1-7b (*Lachnospiraceae*). From left to right, spotting is *Lactobacillaceae* grown in liquid media alone (*Lacto*. only), with NM01_1-7b supernatant (+SUP *Lachno*.), heat-killed (+H-K *Lachno*.), and live (+LIVE *Lachno*.).
