# Supplementary material for: Acids produced by lactobacilli inhibit the growth of commensal Lachnospiraceae and S24-7 bacteria
Source: Gut Microbes. 2022 Mar 10;14(1):2046452. doi: 10.1080/19490976.2022.2046452 (PMC8920129; doi:10.1080/19490976.2022.2046452)
Supplement: Supplemental Material [file KGMI_A_2046452_SM4942.zip › 3.pdf]

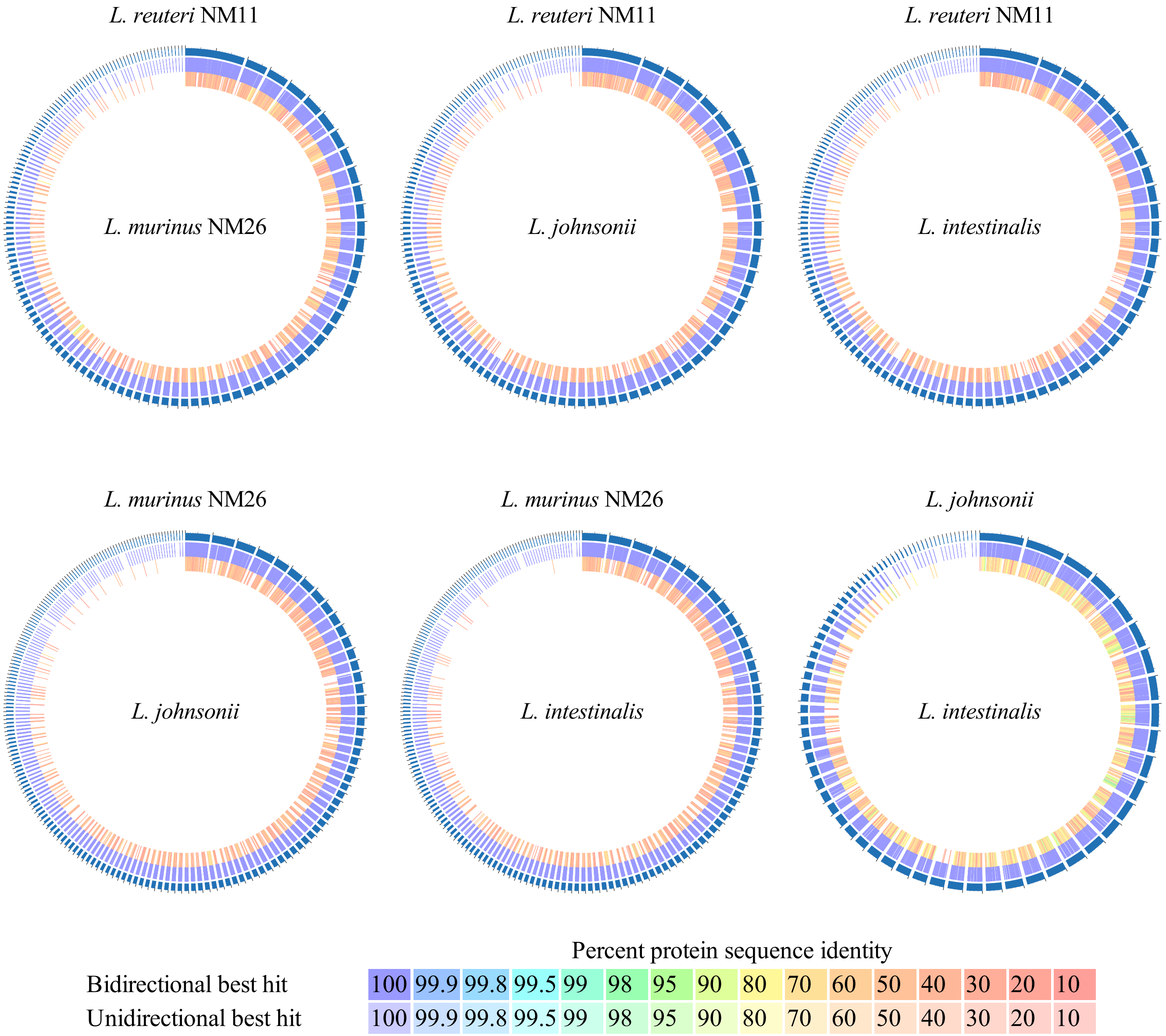

Supplementary Figure 3. Species from the *Lactobacillaceae* family are highly polyphyletic. Genomic comparisons were conducted on PATRIC using bidirectional BLASTP to perform protein sequence-based genome comparisons between each of the four *Lactobacillaceae* species in the CIAMIB. The outermost track shows contigs; the middle track is the 'reference' isolate (labelled above circular tracks); the innermost track is the comparison genome (labelled within circular tracks). As shown in the legend at the bottom, the colour of genes on the innermost track indicates percent protein sequence identity with reference isolate.
