# Supplementary material for: Acids produced by lactobacilli inhibit the growth of commensal Lachnospiraceae and S24-7 bacteria
Source: Gut Microbes. 2022 Mar 10;14(1):2046452. doi: 10.1080/19490976.2022.2046452 (PMC8920129; doi:10.1080/19490976.2022.2046452)
Supplement: Supplemental Material [file KGMI_A_2046452_SM4942.zip › 4.pdf]

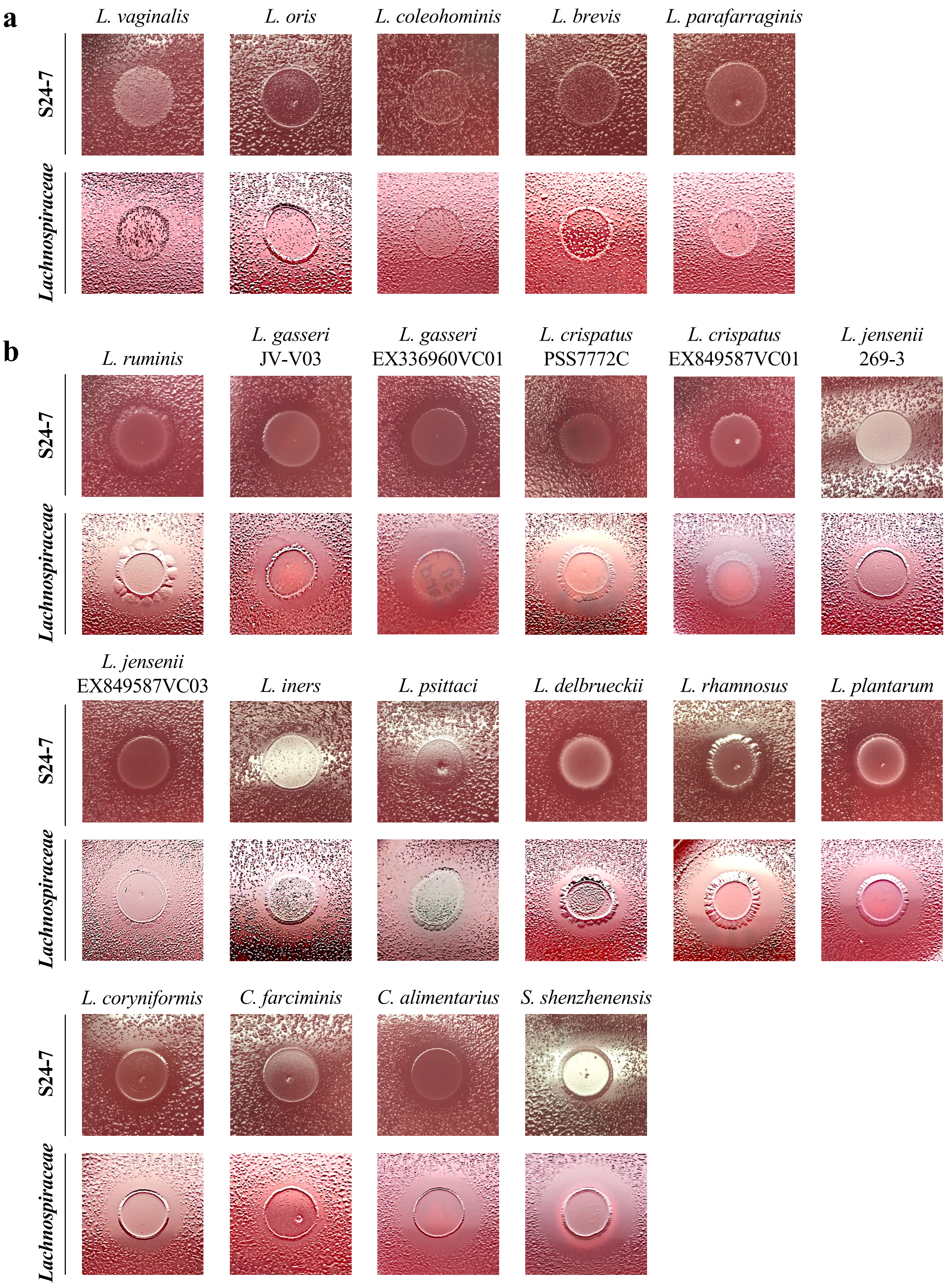

Supplementary Figure 4. Effect of additional *Lactobacillaceae* species on growth of S24-7 and *Lachnospiraceae* representatives. 21 *Lactobacillaceae* strains belonging to 18 additional species were spotted onto lawns of NM74_B14 from the S24-7 group (top rows) and NM01_1-7b from the *Lachnospiraceae* family (bottom rows). Representative images are shown (n = 3). (a) Strains that showed no signs of inhibition of NM74_B14 or NM01_1-7b. (b) Strains that inhibited growth of both NM74_B14 and NM01_1-7b.
