# Supplementary material for: Acids produced by lactobacilli inhibit the growth of commensal Lachnospiraceae and S24-7 bacteria
Source: Gut Microbes. 2022 Mar 10;14(1):2046452. doi: 10.1080/19490976.2022.2046452 (PMC8920129; doi:10.1080/19490976.2022.2046452)
Supplement: Supplemental Material [file KGMI_A_2046452_SM4942.zip › 5.pdf]

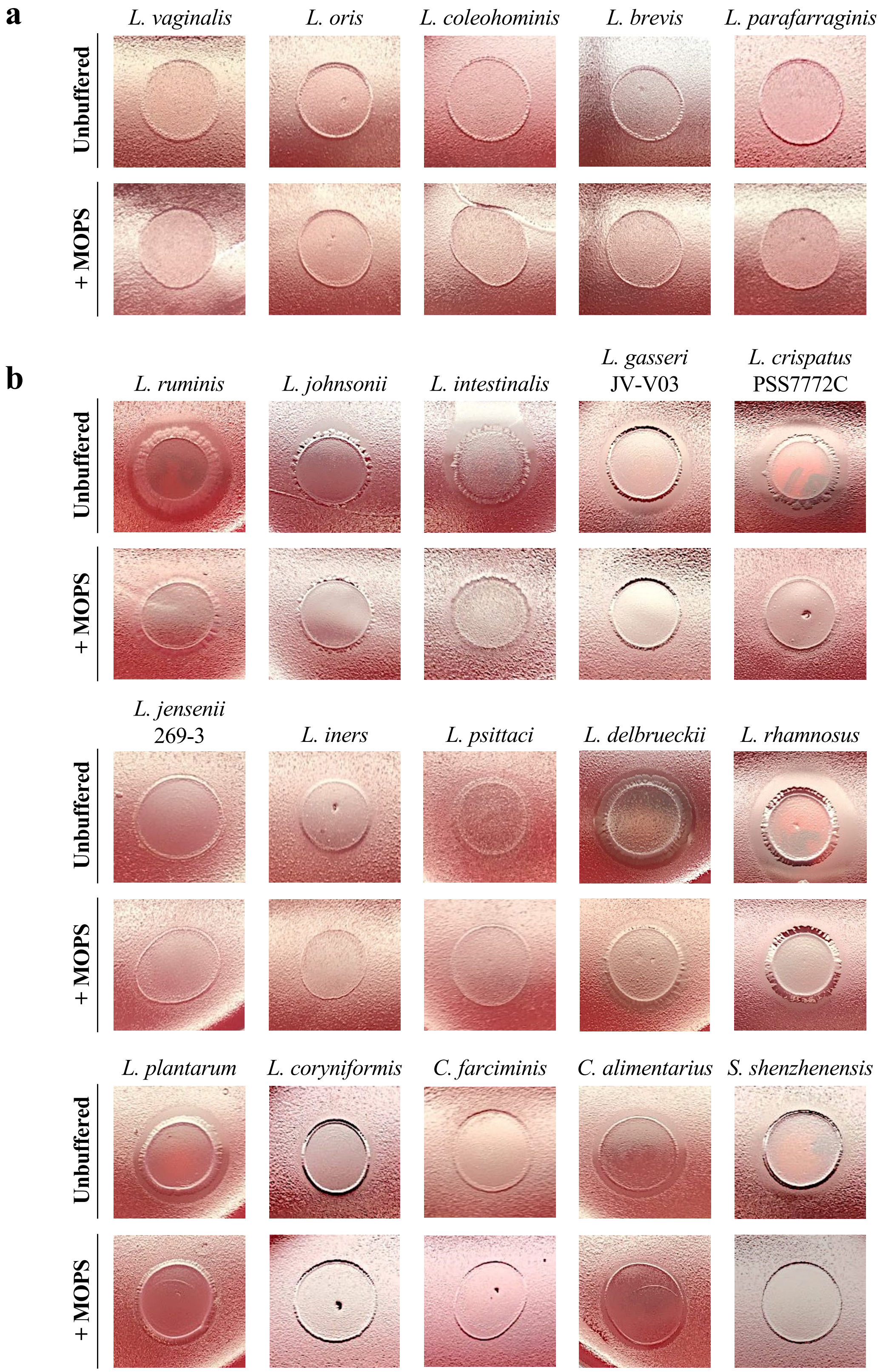

Supplementary Figure 5. The addition of buffer decreases inhibitory effect of *Lactobacillaceae* species against *Lachnospiraceae*. One *Lactobacillaceae* strain of each species spotted onto a lawn of NM01_1-7b (*Lachnospiraceae*). For each *Lactobacillaceae* species, the top spot is on an unbuffered plate while the bottom spot is on a plate supplemented with MOPS buffer at pH 7. Representative images are shown (n = 3). (a) Uninhibitory *Lactobacillaceae* species. (b) Inhibitory *Lactobacillaceae* species.
