# Supplementary material for: Acids produced by lactobacilli inhibit the growth of commensal Lachnospiraceae and S24-7 bacteria
Source: Gut Microbes. 2022 Mar 10;14(1):2046452. doi: 10.1080/19490976.2022.2046452 (PMC8920129; doi:10.1080/19490976.2022.2046452)
Supplement: Supplemental Material [file KGMI_A_2046452_SM4942.zip › 6.pdf]

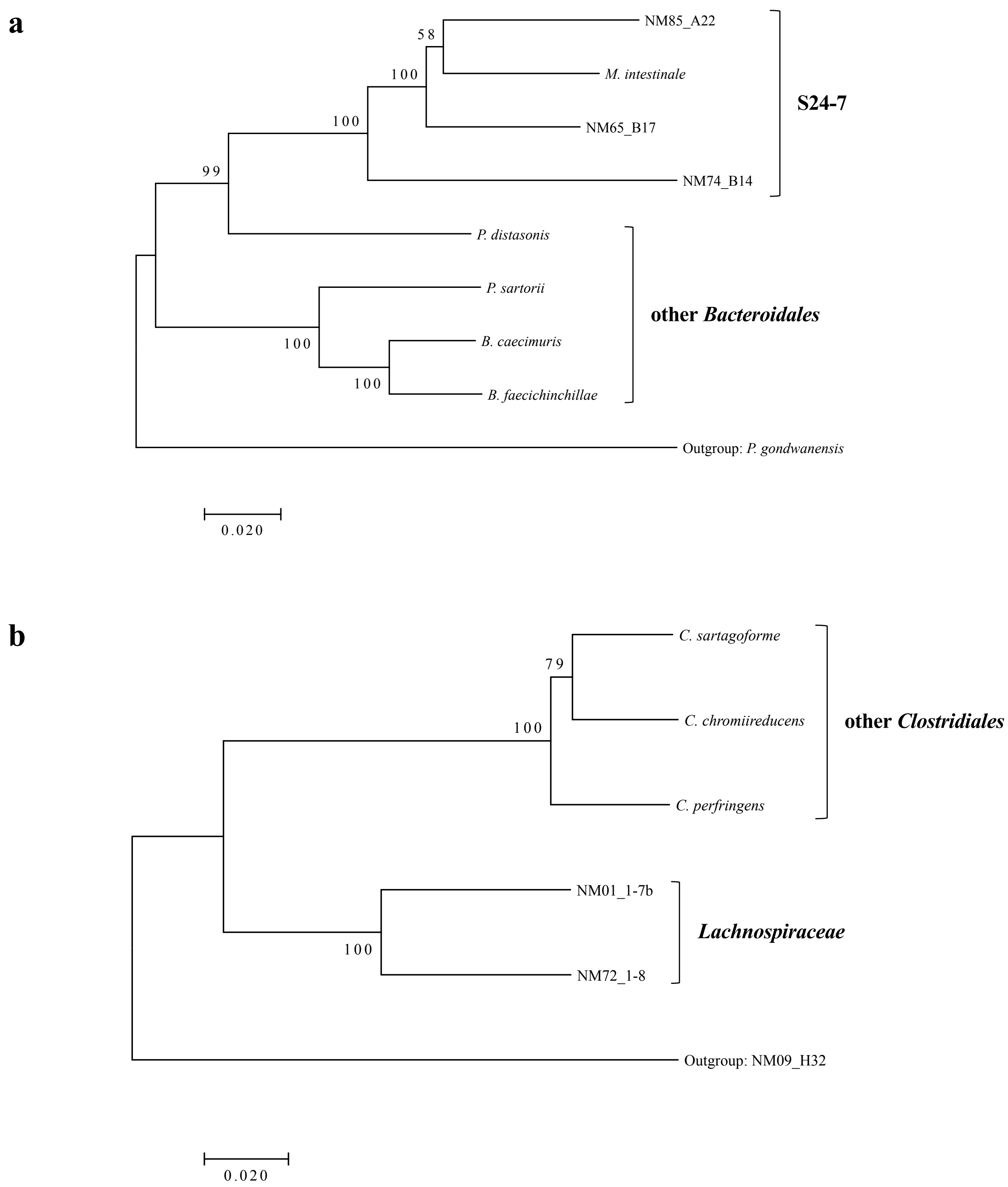

Supplementary Figure 6. Phylogeny of *Bacteroidales* and *Clostridiales* species used in this study. Phylogenetic trees were constructed from 16S rRNA gene sequences. Legends below each tree indicate number of substitutions per site. (a) S24-7 species and other *Bacteroidales* species from the *Bacteroidaceae* family. The outgroup is *Psychroflexus gondwanensis* from the *Flavobacteriales* order. (b) *Lachnospiraceae* species and other *Clostridiales* species from the *Clostridiaceae* family. The outgroup is NM09_H32 from the CIAMIB, belonging to the *Erysipelotrichales* order.
