# Supplementary material for: Acids produced by lactobacilli inhibit the growth of commensal Lachnospiraceae and S24-7 bacteria
Source: Gut Microbes. 2022 Mar 10;14(1):2046452. doi: 10.1080/19490976.2022.2046452 (PMC8920129; doi:10.1080/19490976.2022.2046452)
Supplement: Supplemental Material [file KGMI_A_2046452_SM4942.zip › downloadFromZipFile.pdf]

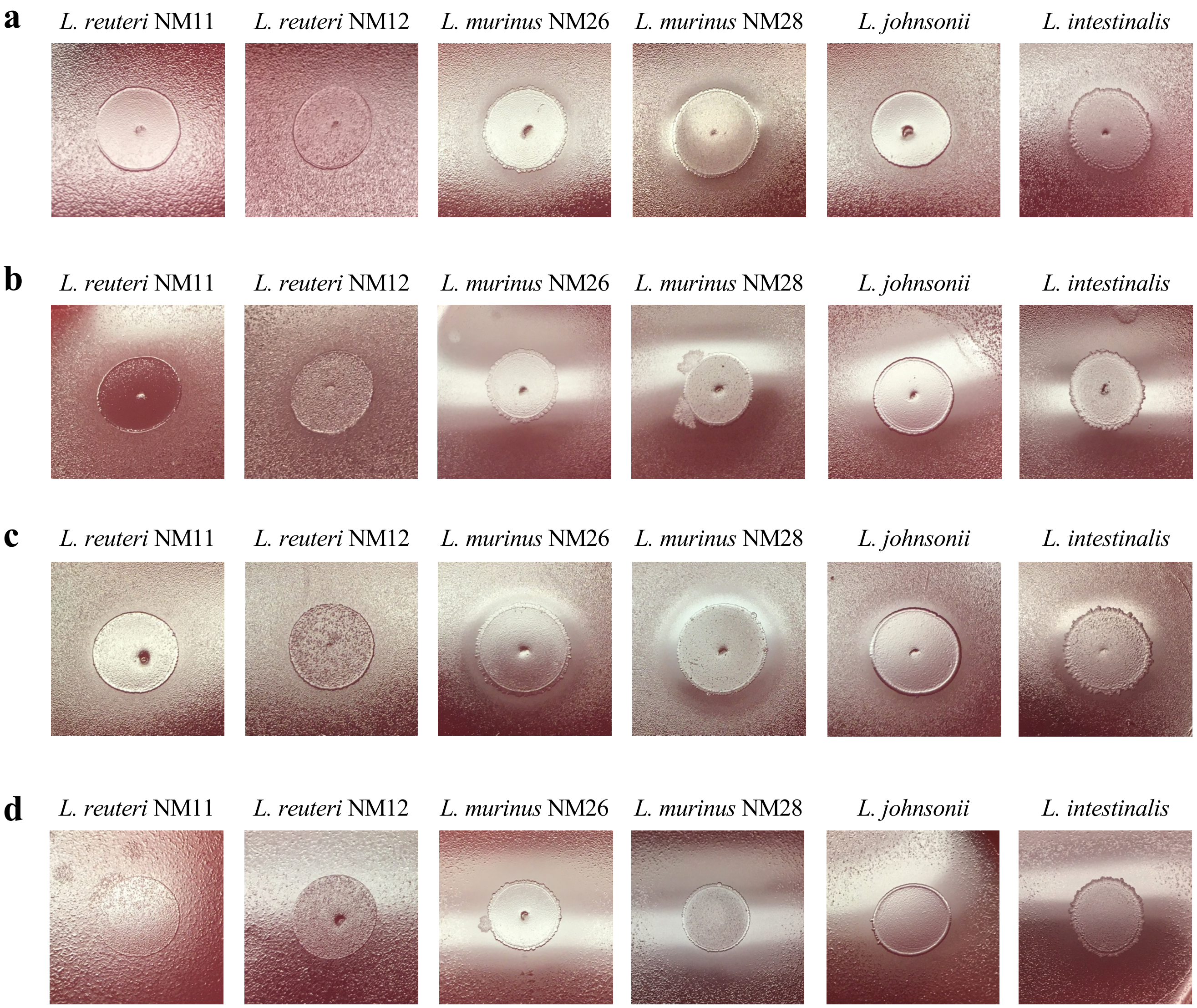

Supplementary Figure 1. Effect of *Lactobacillaceae* species on other S24-7 and *Lachnospiraceae* species in the CIAMIB. Six *Lactobacillaceae* strains belonging to four species spotted onto lawns of (a) *M. intestinale* NM03 (S24-7); (b) NM65_B17 (S24-7); (c) NM86_A22 (S24-7); and (d) NM72_1-8 (*Lachnospiraceae*). Representative images are shown (n = 3).
