# Supplementary material for: Acids produced by lactobacilli inhibit the growth of commensal Lachnospiraceae and S24-7 bacteria
Source: Gut Microbes. 2022 Mar 10;14(1):2046452. doi: 10.1080/19490976.2022.2046452 (PMC8920129; doi:10.1080/19490976.2022.2046452)
Supplement: Supplemental Material [file KGMI_A_2046452_SM4942.zip › Table S4.docx]

Supplementary Table 4. pH and optical density of *Lactobacillaceae* species in liquid media. Values are the average of three independent experiments. Standard deviation is shown for each value.

|  | **Genus** | **Species (strain)** | **pH** | **OD_600_** |
| --- | --- | --- | --- | --- |
| **Heterofermentative**  **(uninhibitory)** | ***Limosilactobacillus*** | ***L. reuteri* (NM11)** | 6.4 ± 0.10 | 0.52 ± 0.07 |
|  |  | ***L. vaginalis*** | 6.6 ± 0.06 | 0.26 ± 0.05 |
|  |  | ***L. oris*** | 6.3 ± 0.00 | 1.00 ± 0.24 |
|  |  | ***L. coleohominis*** | 6.7 ± 0.12 | 0.39 ± 0.18 |
|  | ***Levilactobacillus*** | ***L. brevis*** | 6.7 ± 0.06 | 1.40 ± 0.58 |
|  | ***Lentilactobacillus*** | ***L. parafarraginis*** | 6.7 ± 0.06 | 0.28 ± 0.19 |
| **Homofermentative**  **(inhibitory)** | ***Ligilactobacillus*** | ***L. murinus* (NM26)** | 5.6 ± 0.15 | 0.63 ± 0.05 |
|  |  | ***L. ruminis*** | 5.2 ± 0.10 | 1.86 ± 0.20 |
|  | ***Lactobacillus*** | ***L. johnsonii*** | 6.0 ± 0.15 | 0.72 ± 0.07 |
|  |  | ***L. intestinalis*** | 6.0 ± 0.20 | 1.11 ± 0.45 |
|  |  | ***L. gasseri* (JV-V03)** | 6.2 ± 0.10 | 0.45 ± 0.27 |
|  |  | ***L. crispatus* (PSS7772C)** | 5.8 ± 0.12 | 0.53 ± 0.15 |
|  |  | ***L. jensenii* (269-3)** | 6.3 ± 0.12 | 0.35 ± 0.17 |
|  |  | ***L. psittaci*** | 6.1 ± 0.15 | 0.60 ± 0.15 |
|  |  | ***L. delbrueckii*** | 5.7 ± 0.21 | 1.99 ± 0.45 |
|  | ***Lacticaseibacillus*** | ***L. rhamnosus*** | 5.3 ± 0.06 | 2.65 ± 0.46 |
|  | ***Lactiplantibacillus*** | ***L. plantarum*** | 5.8 ± 0.35 | 2.09 ± 0.40 |
|  | ***Loigolactobacillus*** | ***L. coryniformis*** | 5.6 ± 0.12 | 2.25 ± 0.38 |
|  | ***Companilactobacillus*** | ***C. farciminis*** | 6.0 ± 0.15 | 0.71 ± 0.35 |
|  |  | ***C. alimentarius*** | 6.1 ± 0.32 | 0.52 ± 0.35 |
|  | ***Schleiferilactobacillus*** | ***S. shenzhenensis*** | 6.7 ± 0.06 | 0.23 ± 0.05 |
